# Supplementary material for: Smooth Interpolating Curves with Local Control and Monotone Alternating Curvature
Source: Comput Graph Forum. 2022 Oct 6;41(5):25–38. doi: 10.1111/cgf.14600 (PMC9827861; doi:10.1111/cgf.14600)
Supplement: Supplementary file 1 — Supplement Material [file CGF-41-25-s001.zip › Local-Smooth-Interpolating-MonoCurvature/extern/clothoids/submodules/Utils/ThirdParties/fmt/fmt-8.0.1.zip!/fmt-8.0.1/doc/_templates/search.html]

{#
basic/search.html
~~~~~~~~~~~~~~~~~
Template for the search page.
:copyright: Copyright 2007-2015 by the Sphinx team, see AUTHORS.
:license: BSD, see LICENSE for details.
#}
{%- extends "layout.html" %}
{% set title = \_('Search') %}
{% set script\_files = script\_files + ['\_static/searchtools.js'] %}
{% block extrahead %}
{# this is used when loading the search index using $.ajax fails,
such as on Chrome for documents on localhost #}
{{ super() }}
{% endblock %}
{% block body %}

# {{ \_('Search') }}

{% trans %}Please activate JavaScript to enable the search
functionality.{% endtrans %}

{% trans %}From here you can search these documents. Enter your search
words into the box below and click "search". Note that the search
function will automatically search for all of the words. Pages
containing fewer words won't appear in the result list.{% endtrans %}

{{ searchform('form-inline', True) }}
{% if search\_performed %}

## {{ \_('Search Results') }}

{% if not search\_results %}

{{ \_('Your search did not match any documents. Please make sure that all words are spelled correctly and that you\'ve selected enough categories.') }}

{% endif %}
{% endif %}

{% if search\_results %}

{% for href, caption, context in search\_results %}- {{ caption }}

  {{ context|e }}
{% endfor %}
{% endif %}

{% endblock %}
